# Effectiveness of a Two-Step Testing Algorithm for Reliable and Cost-Effective Detection of *Clostridium difficile* Infection in a Tertiary Care Hospital in Saudi Arabia

**DOI:** 10.3390/medsci7010006

**Published:** 2019-01-08

**Authors:** Mohammed Qutub, Prasanth Govindan, Anupama Vattappillil

**Affiliations:** Department of Pathology and Laboratory Medicine, King Faisal Specialist Hospital and Research Center, Jeddah 21499, Saudi Arabia; qutub.mohammed@kfshrc.edu.sa; (M.Q.); vattappillil@kfshrc.edu.sa (A.V.)

**Keywords:** antibiotic-associated diarrhea, *Clostridium difficile* toxin, enzyme immunoassay, polymerase chain reaction, algorithm-based testing, glutamate dehydrogenase antigen, nucleic acid testing

## Abstract

The aim of this study was to evaluate the effectiveness of a two-step algorithm for the detection of *Clostridium difficile* infection. Setting and Design: A two-step testing algorithm was evaluated for testing stool samples from patients suspected of *Clostridium difficile* infection (CDI). A total of 103 stool specimens were tested using the *C. diff* Quik Chek Complete enzyme immunoassay (EIA) test and the Xpert *C. difficile* PCR test. A two-step algorithm was implemented, and data from 3518 patient samples tested during a two-year period after implementation were analyzed to evaluate the effectiveness. The sensitivity, specificity, and positive and negative predictive values (PPV, NPV) of the Quik Chek Complete EIA test were calculated using the Xpert *C. difficile* PCR test as a reference method. The sensitivity, specificity, PPV, and NPV of the Quik Chek Complete EIA test for *C. difficile* toxin were 46.7%, 100%, 100%, and 91%, respectively. The two-step algorithm, which combined the Quik Chek Complete EIA with Xpert *C. difficile* PCR, improved the sensitivity and also provided rapid detection. When algorithm-based testing was performed daily, there was a 66% reduction in turnaround time compared to batch testing using a lengthy ELISA procedure. Postimplementation data analysis showed that almost 89% of the samples could be reported immediately by initial screening with Quik Chek Complete EIA. Only 11% of the samples gave discrepant results and required PCR confirmation. According to our results, the two-step algorithm is an effective tool for the rapid and reliable detection of toxigenic *C. difficile* from stool samples.

## 1. Introduction

*Clostridium difficile* infection (CDI) is considered the most common cause of hospital-acquired diarrhea [1,2,3]. The latest reports suggest that in the United states of America, CDI has replaced methicillin-resistant *Staphylococcus aureus* (MRSA) as the most common hospital-acquired infection overall [4,5]. The pathogenicity of *C. difficile* is attributed to two toxins: The enterotoxin A, coded by the *tcd A* gene, and the cytotoxin B, coded by the *tcd B* gene [6]. The combined activity of these toxins contributes to the damage caused to the intestinal mucosa and related pathologies. The severity ranges from watery diarrhea to life-threatening conditions like enterocolitis and megacolon [4,7]. A variety of diagnostic techniques are now available for *C. difficile* detection, which include cell culture neutralization, immunological methods, and molecular tests [8]. Culture-based techniques are time consuming, require specialized facilities, and may not be feasible in small and medium-sized laboratories.

The Society for Healthcare Epidemiology of America (SHEA) and the Infectious Diseases Society of America (IDSA) have recommended a two-step testing process for *C. difficile* detection [9]. The two-step testing method includes an initial screening for glutamate dehydrogenase (GDH) antigen and confirmation of all positive cases by toxigenic culture or PCR. This was part of a 2010 guideline that was updated recently in February 2018. The update recommends a multi-step approach to standalone nucleic acid testing (NAT) for *C. difficile* diagnosis [10].

Glutamate dehydrogenase antigen, which is a constitutively synthesized enzyme, is a reliable marker for *C. difficile*, but tests for this antigen cannot differentiate toxigenic and non-toxigenic strains [11,12]. Methods which detect toxins A and B along with the constitutively synthesized GDH antigen offer better clinical sensitivity and specificity. Immunological techniques, including enzyme immunoassays (EIA), are available for the detection of the GDH antigen and the toxins A and B, but often require more hands-on time. Rapid molecular methods have exceptional sensitivity and a short turnaround time (TAT) compared to toxigenic culture, which was once considered the gold standard for *C. difficile* testing [5,13]. However, the relatively higher costs of these assays limit their use, and they are often found to be unaffordable due to budgetary constraints.

The rapid and reliable detection of *C. difficile* significantly reduces the hospital stay and spread of infection. As suggested in recent studies, reducing hospital-associated *C. difficile* and MRSA infections by at least 15% can contribute to cost savings of millions [14,15]. Tertiary care centers that offer oncology, intensive care, and transplant facilities need rapid diagnostic tests for the prompt management of patients. The debilitated patient population in such facilities are at a higher risk of complications associated with *C. difficile* diarrhea [16]. These specialized facilities might receive more samples for *C. difficile* testing with a higher positivity rate compared to other hospitals. Therefore, molecular methods may offer better sensitivity, and some NAT methods can even detect epidemic strains like BI/ NAP1/027, which produces higher-than-usual quantities of toxins A and B.

Various testing algorithms have been proposed in recent times, utilizing immunological assays, culture-based techniques, and molecular methods in different combinations. The utility of these algorithms was dependent on the manpower, infrastructure, and the time taken for completion of the test [17]. Algorithms based on culture-based techniques and conventional molecular methods increased the turnaround time, and required specialized laboratories and skilled technical personnel [18]. The two-test and three-test algorithms, which were developed earlier, improved the clinical specificity and sensitivity, but still had long turnaround times [19,20].

This study evaluated the effectiveness of a two-step testing algorithm for *C. difficile* detection at a tertiary care referral hospital with oncology and transplant facilities serving a patient population particularly vulnerable to CDI. This algorithm proposed an initial screening test using a rapid ELISA method and confirmatory rapid PCR for discordant results. The reliability of this algorithm and efficiency in terms of turnaround time were assessed.

## 2. Materials and Methods

The study was conducted at an internationally accredited tertiary care center in Saudi Arabia, which is a 300-bed tertiary care hospital that processes approximately 1800 reportable *C. difficile* tests per year. The duration of the study was 2 years, from the years 2016–2018, and was approved by the King Faisal Specialist Hospital and Research Centre Institutional Review Board (IRB 2016-27).

### 2.1. Clinical Specimens

A total of 3621 non-formed and liquid stool specimens from patients suspected of CDI were tested in two phases, using two commercial in vitro diagnostic kits for *C. difficile* detection. Samples were tested on the same day of receipt, and an aliquot from each sample was stored at −70 °C for further studies, if required. Only liquid or semisolid stool samples from symptomatic patients were accepted, and formed stools were rejected.

**Figure 1:**
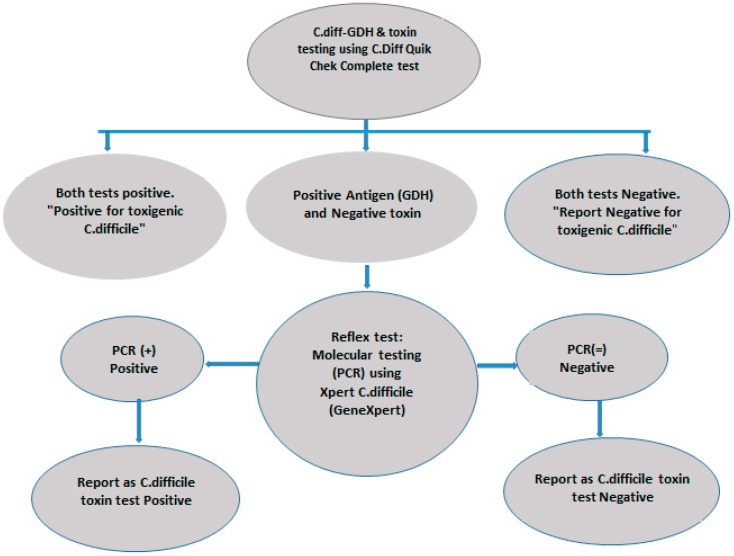
The two-step algorithm for *C. difficile* testing using Quik Chek Complete dual-antigen enzyme immunoassay (EIA) and Xpert *C. difficile* PCR. Stool samples were initially screened using the Quik Chek Complete EIA test. Samples which yielded discrepant results (i.e., positive for antigen and negative for toxin) were tested by PCR. GDH-Glutamate Dehydrogenase.

During phase one, a total of 103 stool specimens were tested using the *C. diff* Quik Chek Complete dual-antigen EIA test and Xpert *C. difficile* GeneXpert PCR, and the results were compared. A two-step algorithm was designed using the Quik Chek Complete EIA and Xpert *C. difficile* PCR to enhance the timeliness of reporting and the judicious use of PCR. This phase focused on evaluating the testing methods and designing the algorithm. Subsequently, the algorithm was implemented for routine *C. difficile* testing. The algorithm is explained in Figure 1.

During phase two of the two-step algorithm, a total of 3518 stool specimens were initially tested using the *C. diff* Quik Chek Complete^®^ dual-antigen EIA test (D-EIA; TechLab, Blacksburg VA, U.S.A.), and the discordant results were confirmed by Xpert *C. difficile* PCR assay following the manufacturer’s instructions. Specimens which provided discordant results (i.e., positive for antigen and negative for toxin) were reflex tested using the Xpert *C. difficile* PCR technique. Data analysis was carried out to test the effectiveness of the new algorithm.

### 2.2. C. difficile Antigen and Toxin Assays

The *C. diff* Quik Chek Complete^®^ dual-antigen EIA (TechLab) is a rapid membrane immune assay measuring the *C. difficile* antigen and toxin. This lateral flow EIA method was performed according to the manufacturer’s instructions. In brief, 25 µL or an equivalent volume of stool specimens was added to a tube containing the diluents and conjugate (TechLab), and the mixture was transferred to the device sample well. After incubation for 15 min at room temperature, the wash buffer and then the substrate (TechLab) were added to the reaction window. The results were read 10 min later. Glutamate dehydrogenase antigen and/or toxins were reported positive if a visible band was seen on the antigen and/or the toxin side of the device display window, respectively. Control dots located in the central portion of the reaction window served as an internal control.

### 2.3. Xpert C. difficile PCR Assay

The Xpert *C. difficile* PCR assay (Cepheid, Sunnyvale CA, U.S.A.) is a multiplex real-time PCR that detects the toxin B gene (*tcdB*), the binary toxin gene (*cdt*), and the *tcdC* deletion at nucleotide (nt) 117. The extraction, amplification, and detection steps take place in different chambers of a self-contained, single-use cartridge containing all the reagents necessary for the detection of *C. difficile* gene targets. The Xpert *C. difficile* PCR was performed according to the manufacturer’s instructions. Briefly, a swab was dipped into the unformed stool specimen container. The swab was placed in a sample reagent and capped. The specimens were vortexed for 10 s, and all the liquid from the sample reagent was transferred to the “S” chamber of the cartridge using a large transfer pipette. Next, reagent 1 was added to chamber 1 of the test cartridge. Finally, reagent 2 was added to chamber 2 of the test cartridge, and the lid was closed. The cartridge barcode was scanned and placed in the GeneXpert instrument. Potential results included the following: Toxigenic *C. difficile* positive and presumptive BI/NAP1/027 negative, toxigenic *C. difficile* positive and presumptive BI/NAP1/027 positive, toxigenic *C. difficile* negative and presumptive BI/NAP1/027 negative, invalid, error, or no results. Testing of specimens with an invalid or error result or no results was repeated once. All the results were available after 45 min.

### 2.4. Data Analysis

Test performance parameters were calculated considering PCR as the reference method. The sensitivity, specificity, positive predictive value, and negative predictive value of the Quik Chek Complete EIA were calculated accordingly. There were no duplicates in the data sets, as repeat samples received within 7 days of initial testing during the same episode of diarrhea were rejected.

## 3. Results

### 3.1. Phase 1

A total of 103 stool specimens were initially tested for *C. difficile* toxin using the Quik Chek Complete EIA and Xpert *C. difficile* PCR. The comparison of Quik Chek CompleteEIA results with the Xpert *C. difficile* PCR assay results is detailed in Table 1.

Of the 103 specimens tested, seven specimens tested positive with both Quik Chek Complete EIA and Xpert *C. difficile* PCR, 88 tested negative with both methods, and eight gave discrepant results (i.e., *C. difficile* antigen positive and toxin negative) by Quik Chek Complete EIA and were retested by Xpert *C. difficile* PCR for confirmation. None of the samples tested negative by Quik Chek Complete EIA assay gave positive results with Xpert *C. difficile* PCR. Hence, the algorithm was implemented for the routine testing of stool samples for *C. difficile* toxin. The sensitivity, specificity, positive predictive value (PPV), and negative predictive value (NPV) of the Quik Chek Complete EIA alone were 46.7%, 100%, 100%, and 91.7%, respectively, and were calculated considering PCR as a reference method.

### 3.2. Phase 2

**Figure 2:**
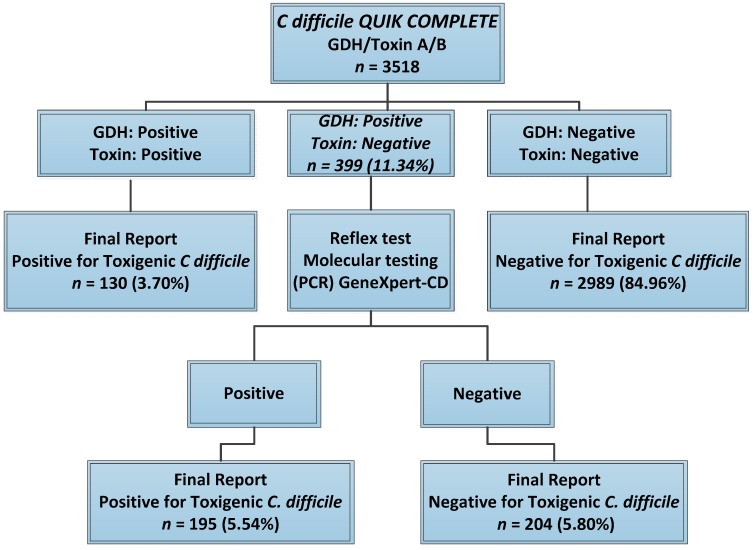
Post-implementation statistics of *C. difficile* testing using the two-step algorithm. Two-year data after implementation of the two-step algorithm showed that almost 89% of the test results were non-discrepant and could be released immediately after using Quik Chek Complete EIA. The rest of the samples (11%) gave discrepant results and required PCR testing for confirmation.

Post-implementation statistical data analysis was carried out to assess the effectiveness of the algorithm. A total of 3518 patient tests performed within two years after implementation of the algorithm were analyzed, and the findings are explained in Figure 2.

Of the 3518 patient results analyzed, almost 89% gave clear non-discrepant results, of which almost 85% were negative and 4% were positive for *C. difficile* toxin using the *C. diff* Quik Chek Complete EIA test. Approximately 11% gave discrepant results and required confirmatory testing with Xpert *C. difficile* PCR. All discrepant results found with the Quik Chek Complete EIA were antigen positive and toxin negative. None of the samples tested negative for antigen and positive for toxin. These findings were identical with the results obtained from the algorithm evaluation during Phase 1.

Prior to the implementation of the two-step algorithm, the turnaround time for *C. difficile* testing was close to 144 h (90th percentile), as the previous method was based on a conventional ELISA which was performed once a week using a lengthy procedure. The postimplementation data showed that the TAT was reduced to less than 48 h (90th percentile), indicating an almost 66% reduction in TAT.

Almost 40% of the total specimens received for *C. difficile* testing were from the medical, surgical, cardiac intensive care, oncology, and transplant units. The overall positivity rate was 9.2%, of which 3.7% was positive by initial screening using Quik Chek Complete EIA, while 5.5% was positive after reflex testing of discrepant results by Xpert *C. difficile* PCR.

## 4. Discussion

The accurate diagnosis of CDI has become increasingly important, given the increased incidence and severity of CDI cases [21]. Since diarrhea is a common complication experienced by debilitated hospitalized patients, the implementation of rapid and sensitive tests for CDI diagnosis is necessary for patient isolation and to investigate diarrhea induced by conditions other than CDI. The proposed two-step diagnostic algorithm has improved efficiency, but continues to depend upon a combination of two assays to improve sensitivity [22]. The goal of the present study was to determine whether PCR could be integrated with EIAs to optimize the detection of toxigenic *C. difficile* while taking advantage of the convenience of rapid immunoassays. As suggested by other investigators [8,23,24], our study used Quik Chek Complete EIA for the initial screening of specimens. This technique, a lateral-flow EIA which could be completed in 30 to 40 min, allowed 85% of specimens to be reported as negative for *C. difficile* or 4% of specimens as toxin-positive, leaving 11% for PCR confirmation due to discrepancy (i.e., antigen-positive and toxin-negative results). The clinical and epidemiological characteristics of these patients were not available to establish the possibility of *C. difficile* colonization, which is a limitation of this study. A discrepancy associated with toxin-positive and antigen-negative results was not observed in our study. This is in agreement with independent studies by Quin et al. and Swindells et al., but in contrast, Sharp et al. experienced this discrepancy with one sample [8,23,24]. Confirmation of discrepant test results by PCR required another hour, but the results were available on the same day of testing.

Algorithm-based testing for the rapid testing of *C. difficile* is gaining importance worldwide, which can considerably reduce the hospital stay and aid in the effective utilization of resources. Toxigenic culture and cell culture cytotoxicity neutralization assay (CCNA) were considered the gold standard until recently, when it was shown that they lacked the desired sensitivity [24,25]. These techniques were labor intensive with a long turnaround time, often limiting their use for routine testing. Quik Chek Complete EIA demonstrated low sensitivity (46.7%) and high specificity (100%), and is comparable to the findings from recent independent studies by Seo et al. and Chung et al. [26,27]. Studies by Sharp et al. and Quin et al. obtained a sensitivity range of 61–73% and a specificity range of 97.3–99.9%. These variations could be attributed to the number of samples tested and differences in the worldwide distribution of *C. difficile* ribotypes [24]. CCNA and toxigenic culture were not performed, and PCR was considered as the reference method in this study owing to its better sensitivity, as suggested by several studies [17,24,28]. This might have contributed to a higher specificity as indicated by Sharp et al. Furthermore, the small sample size of our initial validation is another limitation of our study. Since the sensitivity of GDH antigens is low compared to PCR, the possibility of a missed diagnosis when the Quik Chek Complete EIA method was used as a standalone test cannot be completely ruled out.

Post-implementation statistics from 3518 samples tested after implementation showed a 67% reduction in TAT, from 144 to 48 h, during a two-year period (90th percentile). A two-step labor-intensive ELISA technique was used initially during the pre-implementation period, performed once a week, causing delayed reporting. The Quik Chek Complete EIA assay as well as Xpert *C. difficile* PCR were easier to perform, eliminating the need for dedicated staff, and could be performed daily. This is particularly useful for clinical settings with a higher prevalence of *C. difficile*. Post-implementation data showed 11% discrepant results with Quik Chek Complete EIA, compared to pre-implementation statistics (15.5%). Post-implementation data also showed that 89% of the samples tested did not require confirmatory PCR and were reported on the same day. The cost of testing was effectively reduced as Xpert *C. difficile* PCR was used only for the confirmation of discrepant results. Each Xpert *C. difficile* PCR test was almost four times costlier than Quik Chek Complete dual-antigen EIA tests in terms of material cost. The study shows that Xpert *C. difficile* PCR can be used for real-time confirmation of discrepant *C. difficile* test results, and is a more practical alternative to CCNA and toxigenic culture, as discussed by others [24,28]. In addition, when there are no pre-agreed institutional criteria for *C. difficile* testing, a multi-step approach arbitrated by nucleic acid testing (NAT), rather than NAT alone, has been recommended by the IDSA and SHEA in their recently published updated guidelines [10].

Batch testing using lengthy procedures and three-step algorithms can cause delayed results [20], affecting infection control, institutional cost, and disease-related complications. The algorithm previously in place in our facility was a two-step microliter plate ELISA, which required an initial screening ELISA for GDH, and all GDH-positive samples subsequently requiring a toxin ELISA for confirmation. From a laboratory perspective, this also needed more hands-on time for each specimen and excessive manpower. The two-step algorithm met our workflow needs with satisfactory test results and did not compromise the accuracy of testing. Prompt reporting can also reduce unnecessary isolation and length of hospital stay, mitigating the economic burden of hospital-acquired infection. Our data shows that the majority of the specimens received for *C. difficile* testing were from oncology, intensive care, and transplant units, and these patients required timely diagnosis and management to avoid complications associated with *C. difficile* diarrhea.

In conclusion, the two-step algorithm provides rapid, accurate, and cost-effective detection of toxigenic *C. difficile*. Besides aiding in effective infection control practices and better patient management, it can significantly reduce the cost associated with the length of hospital stay.

## Figures and Tables

**Table 1:** Comparison of Quik Chek CompleteEIA results with Xpert *C. difficile* PCR (prior to implementation of the algorithm; *n* = 103).

		Xpert *C. difficile* PCR		Sensitivity	Specificity	PPV	NPV
		Positive	Negative	%	%	%	%
Quik Chek	Positive	7	0				
				**46.7**	**100**	**100**	**91.7**
Complete EIA	Negative	8 *	88				
			
***95% Confidence interval***				(21.3–73.4%)	(95.9–100%)	100%	(87.3–94.6%)

PPV: Positive predictive value, NPV: Negative predictive value, EIA: Enzyme Immunoassay, PCR: Polymerase chain reaction. The sensitivity, specificity, PPV, and NPV were calculated considering PCR as the reference method. The Quik Chek Complete EIA displayed low sensitivity and high specificity during evaluation. * All discrepant results obtained were GDH antigen positive and toxin negative by the Quik Chek Complete EIA method.
